# *Ortho*‐Carborane‐Derived Halogen‐Bonded Sandwich Complexes

**DOI:** 10.1002/chem.202502693

**Published:** 2025-11-10

**Authors:** Christoph J. Vonnemann, Elric Engelage, Jas S. Ward, Kari Rissanen, Stefan M. Huber

**Affiliations:** ^1^ Fakultät für Biochemie und Chemie Ruhr‐Universität Bochum Universitätsstraße 150 44801 Bochum Germany; ^2^ Department of Chemistry University of Jyväskylä P.O. Box. 35, Survontie 9 B Jyväskylä 40014 Finland

**Keywords:** anion binding, anion recognition, crystallography, halogen bonding, *ortho*‐carboranes

## Abstract

We recently introduced multidentate halogen‐bonding receptors based on C‐iodinated *ortho*‐Carborane moieties. Following up on our results in solution, which indicated a second binding event for sub‐stochiometric concentrations of halide guests, we herein present a systematic co‐crystallization study with halides to further evaluate the halogen‐bonding capacities of these halogen‐bonding catalysts. The obtained solid‐state structures include two rare examples of octahedral coordination via halogen bonding of a halide ion by a pair of tridentate receptors.

## Graphical abstract

A systematic co‐crystallization‐study of a tridentate ortho‐carborane‐based halogen‐bond donor with halides was performed. Several association complexes are evaluated to give further insights into the halogen bond‐strength of this moiety. For iodide, rare octahedral coordination via a sandwich‐like complexation was observed in the solid state when using non‐coordinating and “electron‐rich” cations. This is to our knowledge the first such coordination by halogen‐bonding multidentate receptors.

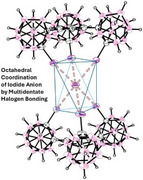

## 1 Introduction

In the field of supramolecular chemistry, anion recognition retains high research interest throughout the years.^[^ ^1^, ^2^, ^3^ ^]^ The halogen bond (XB),^[^ ^4^, ^5^ ^]^ the noncovalent interaction between a positively polarized halide substituent and a Lewis base, has proven to be a powerful tool in this endeavor.^[^ ^6^, ^7^, ^8^, ^9^ ^]^ It has been applied to design co‐crystalline materials of increasing complexity, from simple 1D^[^ ^10^ ^]^ infinite chains to 2D^[^ ^11^ ^]^‐ or 3D^[^ ^12^ ^]^‐architectures. Many such architectures feature halide ions coordinated by multiple XB donors, giving rise to porous networks. In the cavities, different cations have been placed.^[^ ^13^ ^]^ These networks have been utilized, among other things, to tune the electronic properties of some metal coordination complexes.^[^ ^14^, ^15^ ^]^

To gain qualitative insights into the binding situation, single crystal X‐ray diffraction‐based structure determination (SCXRD) of association complexes remains among the most useful of techniques. While threefold coordination of halide ions appears commonly in crystal structures, higher coordination of the halide ions by halogen bonding has been seldomly reported. Though octahedral coordination of halides appears commonly in metal salts and by hydrogen bonding,^[^ ^16^, ^17^ ^]^ such binding by other weak noncovalent interactions remains rare. In 2000, Hawthorne and coworkers reported a sandwiching structure of halides by a mercuracarborand, which they called “anti‐crown binding.”^[^ ^18^ ^]^ The only example of sixfold coordination of bromide by XB donors was to our knowledge reported by Fourmigué in 2013, giving rise to highly symmetrical interpenetrated networks.^[^ ^19^ ^]^ Previously, our group reported up to fivefold coordination of chloride by multidentate perfluorinated XB donors. In those structures, three bonds are coming from a tridentate XB donor and two more from a second molecule (3&2‐motif), resulting in chains of off‐angle penta‐coordinate halide anions and tridentate XB‐donors. For this system, fivefold coordination with an extra XB toward the second receptor was repeatedly observed, while sixfold coordination of the halide by multidentate receptors remained elusive.^[^ ^20^ ^]^

Recently, we introduced a new tridentate halogen‐bonding catalyst based on the *ortho*‐carborane‐moiety.^[^ ^21^ ^]^ In our solution studies, we noted a second binding event for low concentrations of chloride anions, which appeared to have a strong entropically disfavored contribution to its binding energy. We set out to elucidate the observed complex, which we envisioned demonstrated a 2:1 binding of two donors to a single halide ion. We were able to test our hypothesis through systematic co‐crystallization with several noncoordinating salts of chloride, bromide, and iodide (*N.B*. decomposition was observed in the presence of fluoride ions).

## 2 Experimental Section

The halogen‐bonding receptor,^[^ ^21^ ^]^ dimethyl‐imidazolium‐based ionic liquids (MeMIM‐X, X = Cl, Br, I),^[^ ^22^ ^]^ and *tris*‐(dimethylamino) cyclopropenium halides(TDA‐X, X = Cl, Br, I)^[^ ^23^ ^]^ were all synthesized according to previously reported procedures. Pipetting was performed with Hamilton Syringes. Single crystals were grown in 1 ml glass vessels by the combination of 0.2 ml of 10 mM stock solution of **1** in CH_2_Cl_2_ with the corresponding amount of a freshly prepared 100 mM stock solution of guest in CH_2_Cl_2_. The vessel was placed in a 6 ml‐vial filled with 3 ml pentane, capped, and left standing for three days. Suitable crystals for SCXRD were picked directly from the vessel and were immediately analyzed. The single crystal X‐ray data were collected using mirror‐monochromated Cu‐*K*_α_ (*λ* = 1.54184 Å) or Mo‐*K*_α_ (*λ* = 0.71073 Å) radiation on a Rigaku XtaLAB Synergy‐R diffractometer, equipped with either a HyPix‐Arc 100 or a HyPiX6000HE detector, on an Agilent dual‐source Supernova‐diffractometer equipped with either a HyPix‐Arc 100 detector, or on a Rigaku XtaLAB Mini diffractometer equipped with a Mercury375R detector. All structures were solved by intrinsic phasing (SHELXT)^[^ ^24^ ^]^ and refined by full‐matrix least squares on *F* ^2^ using Olex2^[^ ^25^ ^]^ or ShelXle,^[^ ^26^ ^]^ utilizing the SHELXL^[^ ^27^ ^]^ module. Anisotropic displacement parameters were assigned to non‐H atoms and isotropic displacement parameters for all H atoms were constrained to multiples of the equivalent displacement parameters of their parent atoms with *U*_iso_(H) = 1.2 *U*_eq_(CH) or 1.5 *U*_eq_(CH_3_) of their respective parent atoms.^[^ ^28^ ^]^

## 3 Results and Discussion

**Figure 1:**
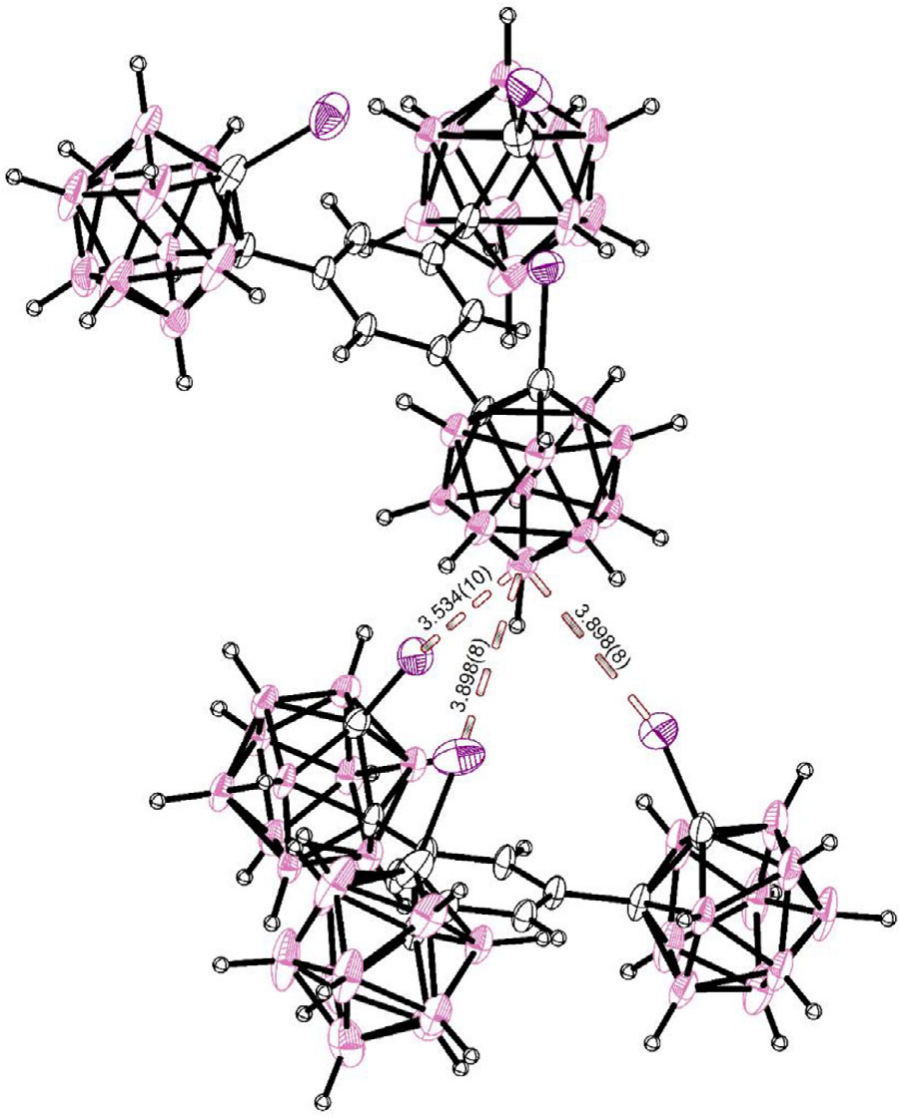
SCXRD‐structure of the halogen‐bonding receptor (**1**), showing close contact of the tridentate iodine binding site toward the electron rich domain of a neighboring carborane moiety. The combined van‐der‐Waals‐radii of boron and iodine are 3.90 Å.^[^ ^29^ ^]^ CH_2_Cl_2_ solvent molecules omitted for clarity. Thermal ellipsoids at 50% probability level.

To begin our study, single crystals of the pure tridentate receptor (**1**) were grown (see Figure 1). For this, slow evaporation of a CH_2_Cl_2_ solution of the complex was sufficient, though vapor‐diffusion from CH_2_Cl_2_ solution of the complex with pentane as the antisolvent‐ yielded identical crystals. We obtained orthorhombic crystals containing one CH_2_Cl_2_ solvate molecule in its asymmetrical unit. To our surprise, all three iodine substituents in **1** of the freely rotating *ortho*‐carborane‐subunits point in the same direction toward the most electron‐rich B(9) of a neighboring carborane cluster, with all carborane‐C‐C‐I‐planes orthogonal (90.4° and 93.1(5)°) to the central benzene‐scaffold. There are in fact close contacts of the iodine‐substituents with the B(9), approaching as close as 90.6% of their combined van‐der‐Waals‐radii^[^ ^29^ ^]^ (*R*_XB_ **= 0.906), while the other two symmetrical iodine substituents point toward the middle of the B(9)─B(12)‐bond, the most electron rich surface‐region of the cluster. The high linearity of the carbon‐iodine‐boron‐contact (173.5°) further indicates that the carborane‐moiety acts as a XB acceptor.

**Figure 2:**
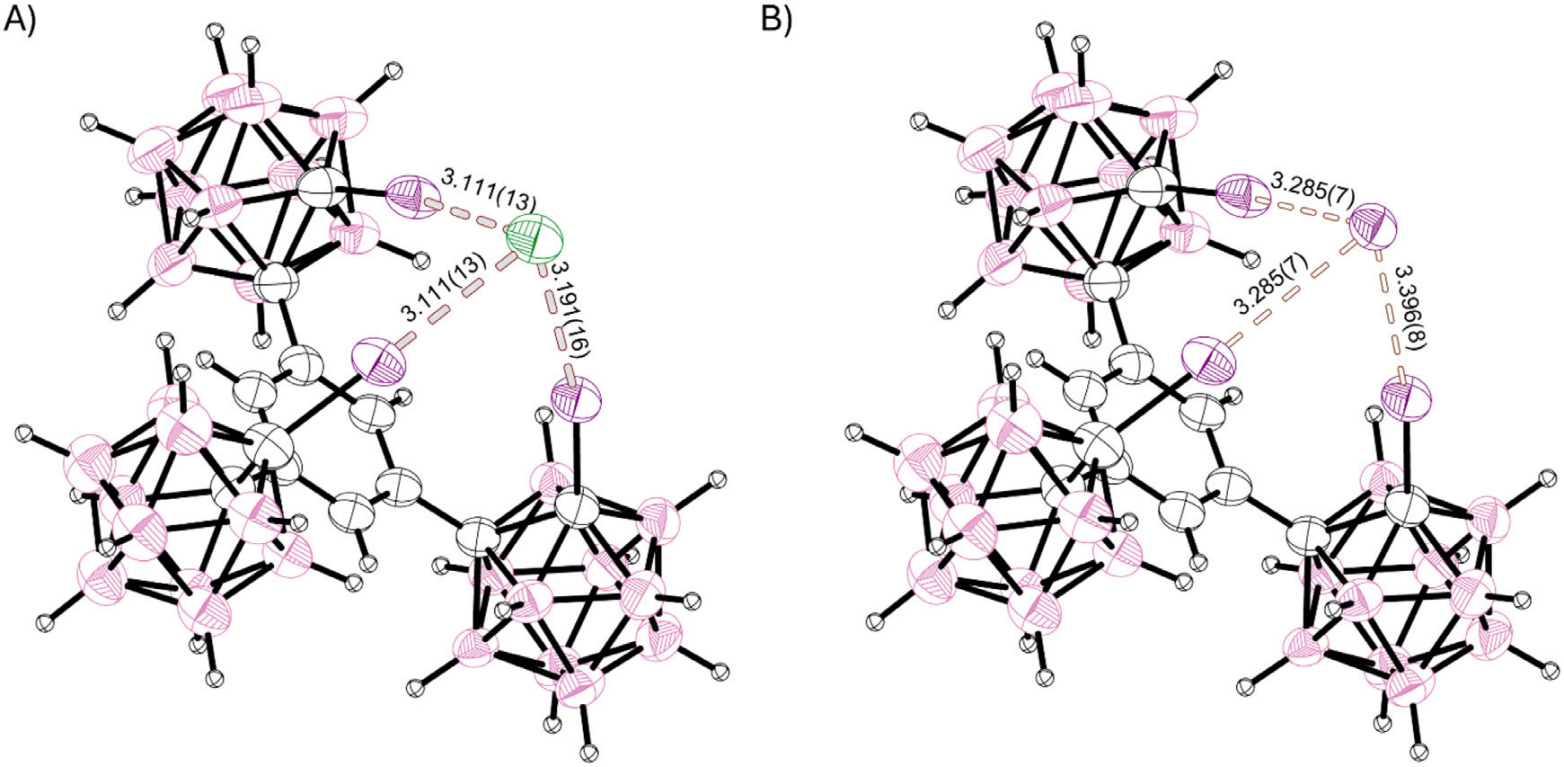
Partial SCXRD‐structures of the halogen‐bonding receptor co‐crystallized with less than one equivalent of TBA‐Cl. The crystal (structure **2**) is isostructural to the previously reported bromide structure. A) shows the chloride guest (68% occupancy), and B) depicts the iodide (32% occupancy) attributed to scrambling of the anion. The combined van‐der‐Waals‐radii of chlorine and iodine are 3.73 Å, and for iodine and iodine 3.96 Å.^[^ ^29^ ^]^ TBA‐counterions omitted for clarity. Thermal ellipsoids at 50% probability level.

Following up on our published^[^ ^21^ ^]^ crystal structure of the receptor with tetrabutyl ammonium bromide (TBA‐Br), and seeing that we only detected a second binding event in solution with sub‐stochiometric amounts of chloride, we were interested in cocrystals with TBA‐Cl. Indeed, under the same conditions, isostructural monoclinic cocrystals were grown (Structure **2**). Interestingly, the obtained crystal only seems to contain 68% chloride, while 32% of the halide's position is occupied by iodide (see Figure 2). This is only reasonably explained by scrambling or partial decomposition of the catalyst in low concentrations of chloride ions, liberating trace iodide. In our earlier solution‐phase studies, we did not detect any such decomposition. Binding of both the chloride and the iodide ion in the crystal (**2**) is equally strong as for the bromide ion. The two symmetry‐equivalent bonds are slightly shorter (*R*_XB_ **= 0.834, ∡= 171.6° for chloride, *R*_XB_ **= 0.839, ∡= 171.1° for iodide) than the third bond (*R*_XB_ **= 0.855, ∡= 168.8° for chloride, *R*_XB_ **= 0.858, ∡= 174.8° for iodide), which is exactly in line with the bonds in the bromide‐crystal (*R*_XB_ = 0.837 and 0.858, ∡= 169.7° and 172.7°, respectively).

**Figure 3:**
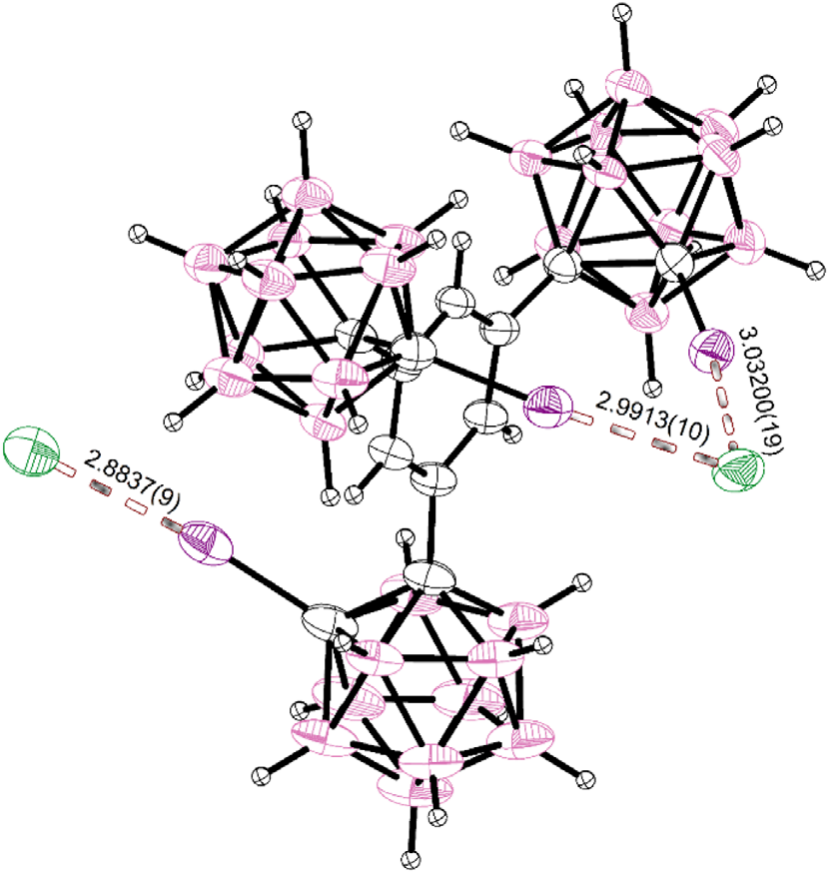
SCXRD‐structure (structure **3**) of the halogen‐bonding receptor co‐crystallized with two equivalents of TBA‐Cl, assuming a mixed bidentate and monodentate *anti*‐conformation. The combined van‐der‐Waals‐radii of chlorine and iodine are 3.73 Å.^[^ ^29^ ^]^ TBA‐counterions omitted for clarity. Thermal ellipsoids at 50% probability level.

More curiously, the above‐mentioned scrambling was not observed in crystals grown according to the same procedure with higher equivalents of TBA‐Cl (Structure **3**). Instead, in the resulting monoclinic crystal the tridentate receptor assumes an *anti*‐conformation (see Figure 3, again demonstrating the free rotation of the iodinated *ortho*‐carborane moieties) with two iodo‐carborane‐moieties coordinating one chloride ion in a bidentate fashion (*R*_XB_ **= 0.802, ∡= 172.2(1)° and *R*_XB_ **= 0.813, ∡= 172.2(1)°), and the third binding a second chloride ion with a very strong XB (*R*_XB_ **= 0.773, ∡= 174.9(1)°).

**Figure 4:**
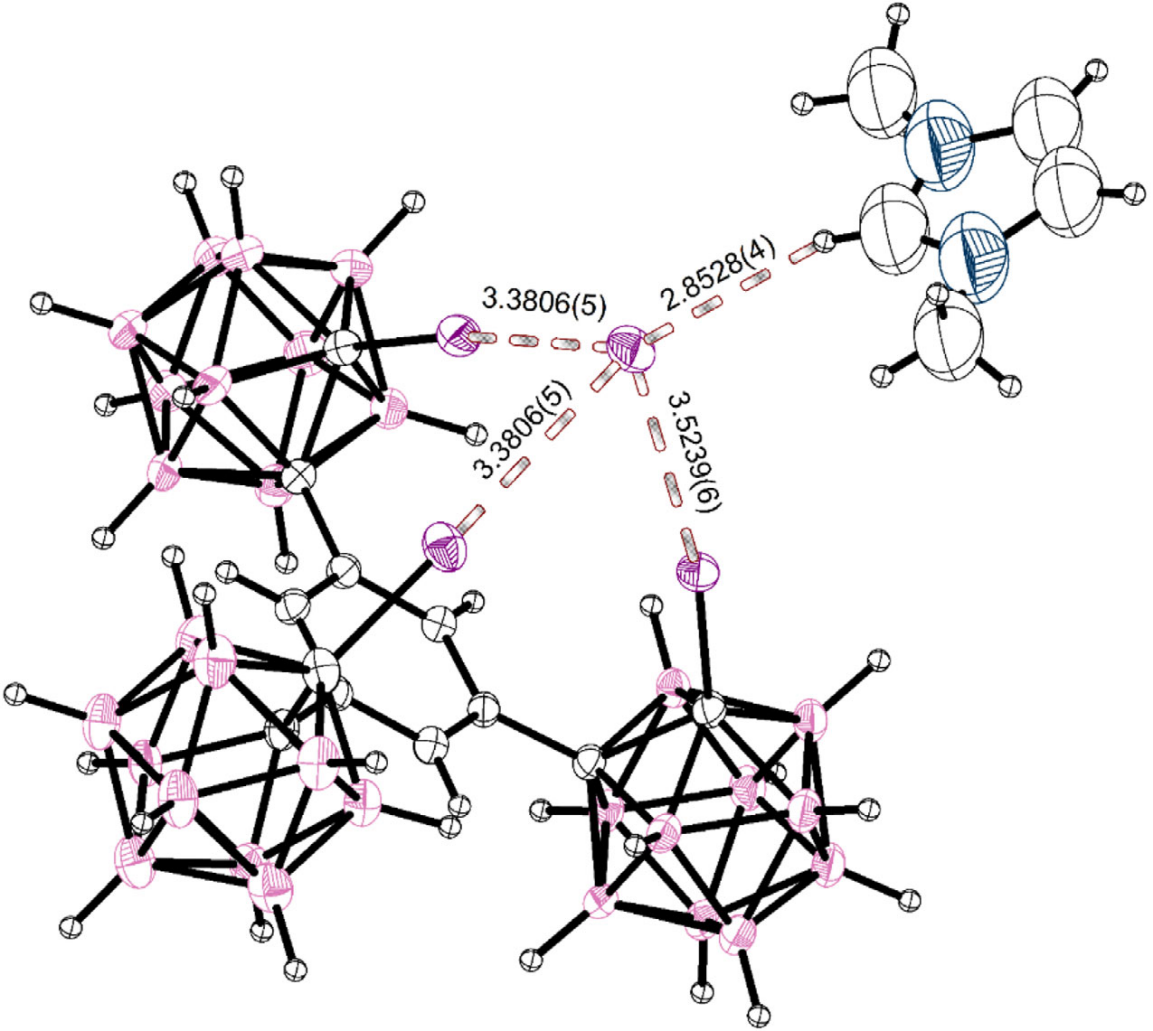
SCXRD‐structure (structure **5**) of the halogen‐bonding receptor co‐crystallized with one equivalent of MeMIM‐I. The combined van‐der‐Waals‐radii of iodine and iodine 3.96 Å, and of iodine and hydrogen are 3.18 Å.^[^ ^29^ ^]^ The MeMIM‐cation exhibits heavy disorder. Thermal ellipsoids at 50% probability level.

In all cases, the TBA counterions are located closely around the halide ions in the obtained lattices, preventing further complexation of the ions. To crystallize the postulated 2:1 association complex, we then attempted co‐crystallization with salts containing either dimethyl‐imidazolium‐based ionic liquids (MeMIM‐X) or “electron rich cations”, such as the *tris*‐(dimethylamino)cyclopropenium (TDA‐X) cation,^[^ ^30^ ^]^ seeking to exploit its tendency to separate the charges in cocrystals. When applied in a 1:1 stoichiometry, the receptor crystallizes with MeMIM‐Cl well, though we only observed **1** and the iodide ion in the obtained lattice due to disorder of the cation. Fitting any remaining chloride ion to that position always yielded higher errors in the refinement of the structure (see Supporting Information for details). The same crystal structure was also obtained when the receptor was crystallized with pure MeMIM‐I (shown in Figure 4).

In the obtained crystals of the receptor with MeMIM‐I (**5**), again half a receptor molecule forms the asymmetrical unit of the lattice. There are two symmetry‐equivalent strong halogen bonds (*R*_XB_ **= 0.854 and ∡= 172.6(1)°) and a slightly longer third contact (*R*_XB_ **= 0.890 and ∡= 170.3(1)). The cation is situated – hydrogen bonding (*R*_HB_ **= 0.933) to the iodide ion – in opposition to the two symmetrical bonds. It is taking almost the same position as the TBA‐ions in those crystals. When we grew crystals of MeMIM‐Br and **1**, again the same structure (**4**) was obtained, though in this case a 45% scrambling of iodide into the halide position was observed. Additionally, we noticed bad resolution of the MeMIM‐cation, with crystallographic datasets repeatedly failing to yield sensible structures. As a result of this we had to utilize Platon Squeeze,^[^ ^31^ ^]^ recovering electron density for 332 electrons in solvent‐accessible voids, fitting to the heavily disordered cations and some unknown solvates (possibly up to 3 molecules of water). We did not find any solvates in the more well‐resolved isostructural crystals, though.

**Figure 5:**
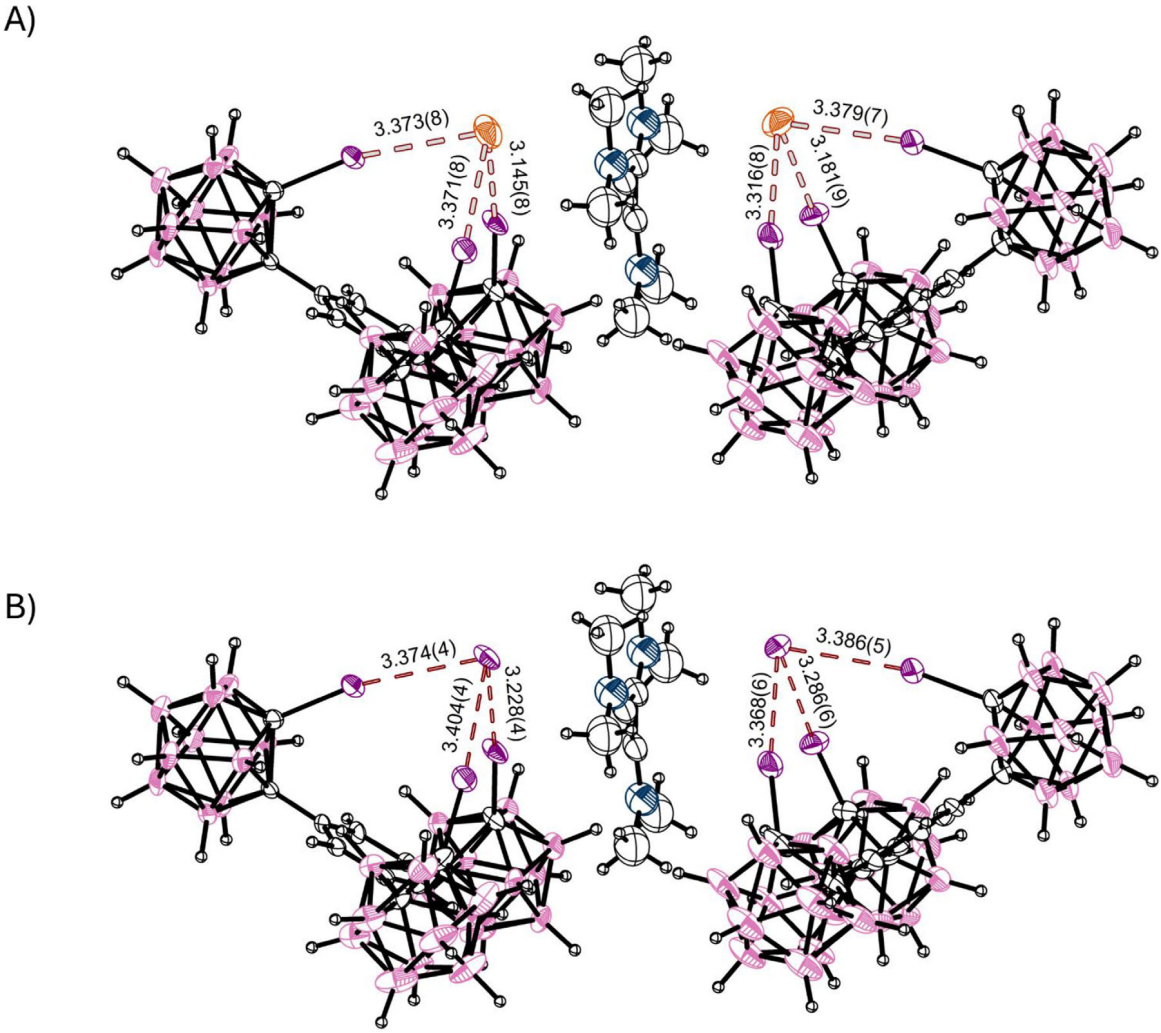
SCXRD‐structures (structure **6**) of the halogen‐bonding receptor **1** co‐crystallized with one equivalent of TDA‐Br. The combined van‐der‐Waals‐radii of bromine and iodine are 3.73 , and of iodine and iodine 3.96 Å.^[^ ^29^ ^]^ A) shows the bromide guests, B) depicts the scrambled in iodide taking 40% (left) and 50% (right) of the halide's position. The TDA‐cation shown, sandwiched between the halides, exhibits heavy disorder, a second nonparticipating counterion is omitted for clarity. Thermal ellipsoids at 50% probability level.

When applying equimolar amounts of TDA‐Cl, no suitable crystals were obtained, instead only yielding microcrystalline TDA‐Cl samples. Utilizing TDA‐Br instead, the expected 1:1 complex (**6**) was obtained, though again exhibiting significant scrambling (See Figure 5). In the obtained crystal, disorder leads to two slightly different binding situations, though they are exhibiting similar XBs. A disordered TDA‐cation is positioned sandwiched in between two halide‐bearing receptors (3:2 Br:I and 1:1 Br:I). Both binding events show one shorter bond (R*_XB _* = 0.85, 0.84, ∡= 171.2(2)°, 173.3(2)°) toward the bromide and two almost equal bonds (averages *R*_XB_ **= 0.91, 0.90, ∡= 168.5(2)°, 169.1(2)°), their difference being smaller than their combined errors. Toward the iodide, the bonds are again as expected and similar to those previously observed, with the same bond being slightly shorter (*R*_XB_ **= 0.83, 0.82, ∡= 171.3(2)°, 173.3(2)°).

**Figure 6:**
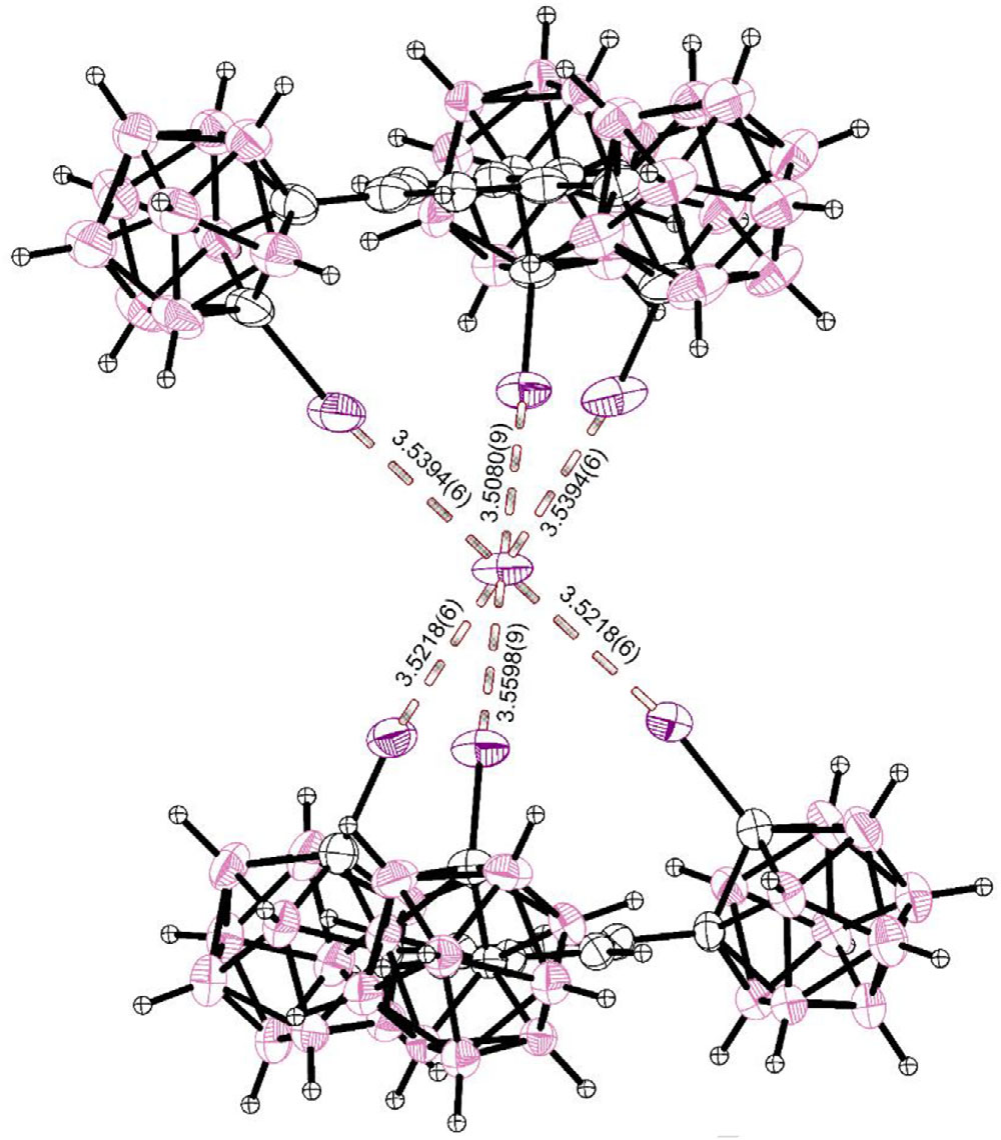
SCXRD‐structure (structure **7**) of two molecules of **1** co‐crystallized with half an equivalent of MeMIM‐I. Two receptors are sandwiching a single iodide ion, exhibiting six halogen bonds of similar strength across the two tridentate binding environments. The combined Van‐der‐Waals‐radii of iodine and iodine are 3.96 Å. The cation could not be resolved due to heavy disorder. Thermal ellipsoids at 50% probability level.

Due to the observed scrambling, we focused on the iodide anion.^[^ ^32^ ^]^ When co‐crystallizing the receptor with half the amount of corresponding MeMIM‐I, crystals of the adduct (**7**) were obtained (See Figure 6). The iodide anion was coordinated in an octahedral sandwich‐like fashion, with two symmetry‐defined equal bonds coming from one receptor each. The bond lengths, all between 3.5080(9) Å (*R*_XB_ **= 0.886) and 3.5598(9) Å (*R*_XB_ **= 0.899) and bond‐angles between 170.9(2)° and 172.7(2)° (mean 171.9(3)°) are right inside the observed parameters from the 1:1 association complex, showing two effectively equal‐strength binding events being present. We envision the same complex forming in our observations in solution for the case of the much more charge‐dense chloride at low concentrations.

In the crystal, the counter‐cations were again heavily disordered and not properly assignable, in the same fashion as the MeMIM‐cation tended to be in 1:1 association‐complexes. As the sandwich structure resolved well, we accounted for the counterion using Platon Squeeze, recovering 560 electrons per unit cell, which corresponds to the four counterions per unit cell and possibly 8.3 molecules of disordered CH_2_Cl_2_ solvate or other solvates (such as water) in solvent accessible voids.

**Figure 7:**
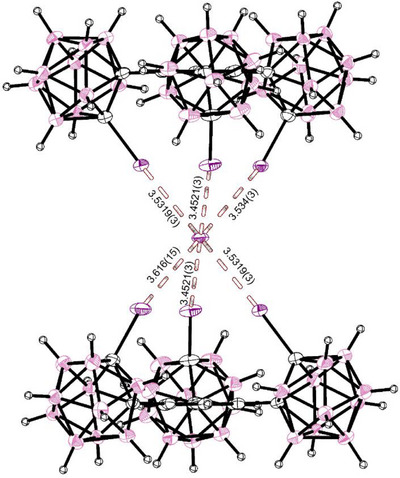
SCXRD‐structure (structure **8**) of two molecules of **1** co‐crystallized with half an equivalent of TDA‐I. Two receptors are sandwiching a single iodide ion, exhibiting six halogen bonds of similar strength across two tridentate bindings, one Carborane‐I─I‐Bond being slightly disordered (69:31). The combined van‐der‐Waals‐radii of iodine and iodine are 3.96 Å. The counterion, as well as the disordered methylene chloride solvate is omitted for clarity. Thermal ellipsoids at 50% probability level.

When co‐crystallized with TDA‐I instead, a structure was obtained with the iodide ion sitting at an inversion center (See Figure 7). The obtained structure is very similar to the MeMIM‐I‐complex. Again, two receptors sandwich an iodide ion with slightly shorter overall bond lengths between 3.4521(3) Å (*R*_XB_ **= 0.872) and 3.616(15) Å (*R*_XB_ **= 0.913) with a mean of 3.514(3) Å (*R*_XB_ **= 0.887), though the furthest bonding iodine appears slightly disordered. The bond angles are slightly less linear with angles between 163.7(6)° and 171.7(1)° (mean 168.6(4)°).

In all obtained crystal structures, a particular binding pattern was observed. Two nearly equivalent (or truly equivalent due to symmetry) shorter bonds bind the halide in a bidentate fashion, with a third contact of slightly longer bond length (Δ*R*_XB_ **= 0.01–0.04) completing the tridentate motif. In contrast to topographically similar tridentate perfluorinated XB donors,^[^ ^20^ ^]^ the central benzene core does not exhibit any significant distortion in any of the obtained solid‐state structures. This highlights the superior compatibility of the bite angle toward the coordinated halide ions for these systems. Small size differences are instead compensated by the rotation of the *ortho‐*carborane moiety, which should be energetically much more favorable, while the linearity of the bond angles is still somewhat preserved (159.9°–174.9° in the carborane‐based receptors vs. 168°–177° for fluorinated receptors). The fact that two very similar 2:1 complexes with hexacoordinated iodide were obtained for two structurally quite different cations speaks to the robustness of this motif.

## 4 Summary and Conclusion

In total, we presented seven cocrystals of the halides chloride, bromide, and iodide with our new *ortho*‐carborane‐based halogen‐bonding receptor utilizing different noncoordinating counter‐ions. Most importantly, a sandwich‐like structure was obtained, which we first postulated from our solution‐based studies based upon a secondary interaction at low equivalents of halide ions. In this structure, the iodide ion was octahedrally coordinated, reports of which are still very rare for nonmetalated systems. To the best of our knowledge, this represents only the second case in which such an association was realized with halogen bonding. We anticipate that this “engulfing” complexation of iodide could form the basis of future applications, for example, for halide‐abstraction reactions or for the solubilization of iodide salts.

## Supporting Information

Supporting Information available containing crystallographic details.

## Conflict of Interest

The authors declare no conflict of interest.

## Author Contributions

C.J.V. and S.M.H. conceived the study. C.J.V. performed the synthesis. C.J.V., E.E., and J.S.W. performed the crystallographic studies (data collection, structure solution, and refinement). K.R. advised on crystallography. C.J.V and S.M.H wrote the manuscript draft, and all authors contributed to the writing process. S.M.H. provided guidance throughout the project.

## Supporting information



Supporting Information

Supporting Information

## Data Availability

Data available in article supplementary material.
