# Supplementary material for: Ortho‐Carborane‐Derived Halogen‐Bonded Sandwich Complexes
Source: Chemistry. 2025 Nov 10;31(71):e02693. doi: 10.1002/chem.202502693 (PMC12734684; doi:10.1002/chem.202502693)
Supplement: Supplementary file 1 — Supporting Information [file CHEM-31-e02693-s001.pdf]

Ortho-Carborane-Derived Halogen Bonded Sandwich Complexes

Christoph J. Vonnemann, Elric Engelage, Jas S. Ward, Kari Rissanen, and Stefan M. Huber*

Supporting Information

Table of Contents

Crystal Structure Data 2

Structure of the tridentate receptor 2

Cocrystal of the tridentate receptor and TBA-Cl 3

Cocrystal of the tridentate receptor and 2 TBA-Cl....................4

Cocrystal of the tridentate receptor and MeMIM-Br 5

Cocrystal of the tridentate receptor and TDA-Br....................7

Cocrystal of the tridentate receptor and ½ MeMIM-I 8

Cocrystal of the tridentate receptor and ½ TDA-I....................9

NMR 10

Crystal Structure Data

Structure of the tridentate receptor

Table S1 Crystal structure data.

**Identifier**	1
**Formula**	$C_{13}H_{35}B_{30}Cl_2I_3$
**Molecular Mass [gmol^{-1}]**	967.31
**Crystal Habit**	Yellow block
**Crystal Dimensions**	0.04 × 0.05 × 0.08
**Crystal System**	Orthorhombic
**Space Group**	*Pnma*
**a [Å]**	16.1286(2)
**b [Å]**	19.0653(3)
**c [Å]**	11.9084(1)
**α [°]**	90
**β [°]**	90
**γ [°]**	90
**Volume [Å^3]**	3661.79(8)
**Z**	4
**Density [gcm^{-3}]**	1.75451
***F*(000)**	1824.0
**μ [mm^{-1}]**	21.521
**T [K]**	121
**Completeness / Θ_{max}**	99.9 / 74.5
**Total Reflections**	44247
**Unique Reflections**	3851
**Reflections [Io > 2σ(Io)]**	3346
**R_{int}**	0.0530
**Goodness of Fit**	1.045
**Parameters**	229
**Restraints**	0
**Largest Peak/Hole (eÅ^{-3})**	2.58 / -1.65
**R_1 (observed/all)**	0.0572 / 0.0642
**wR_2 (observed/all)**	0.1624 / 0.1687
**CCDC Number**	2483324

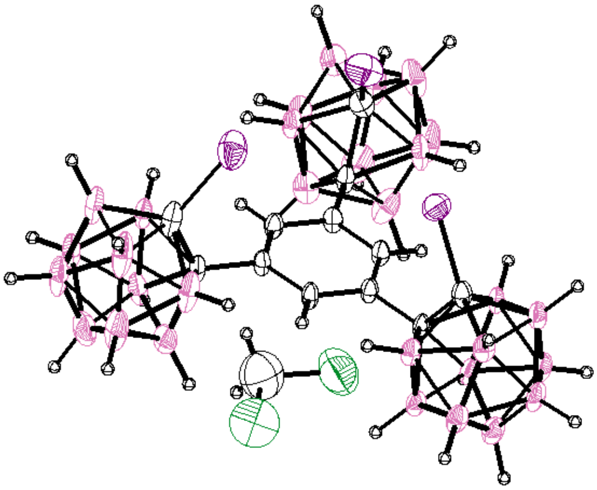

Cocrystal of the tridentate receptor and TBA-Cl

Table S2 Crystal structure data.

**Identifier**	2
**Formula**	$C_{28}H_{69}B_{30}Cl_{0.68}I_{3.32}N$
**Molecular Mass [gmol^{-1}]**	1189.55
**Crystal Habit**	Yellow plate
**Crystal Dimensions**	0.02 × 0.07 × 0.22
**Crystal System**	Monoclinic
**Space Group**	*I*2/*m*
**a [Å]**	17.3020(3)
**b [Å]**	18.5943(3)
**c [Å]**	18.6086(3)
**α [°]**	90
**β [°]**	105.254(2)
**γ [°]**	90
**Volume [Å^3]**	5775.81(17)
**Z**	4
**Density [gcm^{-3}]**	1.368
***F*(000)**	2326
**μ [mm^{-1}]**	14.526
**T [K]**	170
**Completeness / Θ_{max}**	99.8 / 74.492
**Total Reflections**	36629
**Unique Reflections**	6092
**Reflections [Io > 2σ(Io)]**	5120
**R_{int}**	0.0434
**Goodness of Fit**	1.109
**Parameters**	371
**Restraints**	149
**Largest Peak/Hole (eÅ^{-3})**	0.97 / -1.01
**R_1 (observed/all)**	0.0416/0.0493
**wR_2 (observed/all)**	0.1126/0.1178
**CCDC Number**	2483325

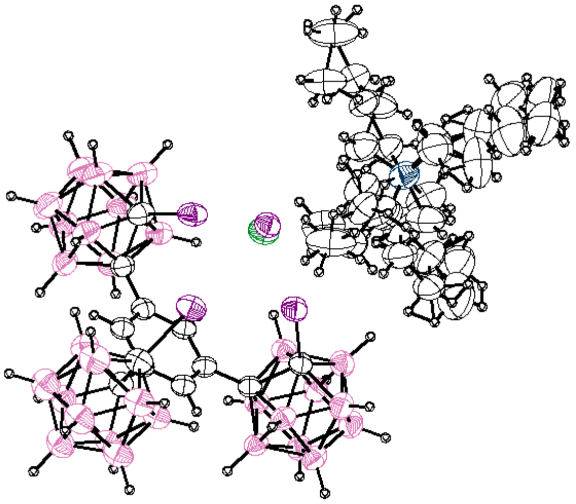

* Platon Squeeze was used to account for the unknown solvate(s) present, and no correction has been made to the formula, density, *etc*.

Cocrystal of the tridentate receptor and 2 TBA-Cl

Table S3 Crystal structure data.

**Identifier**	3
**Formula**	$C_{44}H_{105}B_{30}Cl_2I_3N_2$
**Molecular Mass [gmol^{-1}]**	1438.19
**Crystal Habit**	Colorless needle
**Crystal Dimensions**	0.04 × 0.04 × 0.14
**Crystal System**	Monoclinic
**Space Group**	$P2_1/n$
**a [Å]**	16.1198(1)
**b [Å]**	19.9751(2)
**c [Å]**	23.3294(2)
**α [°]**	90
**β [°]**	91.546(1)
**γ [°]**	90
**Volume [Å^3]**	7509.2(1)
**Z**	4
**Density [gcm^{-3}]**	1.272
***F*(000)**	2904
**μ [mm^{-1}]**	10.664
**T [K]**	170
**Completeness / Θ_{max}**	99.9 / 67.684
**Total Reflections**	96303
**Unique Reflections**	15302
**Reflections [Io > 2σ(Io)]**	12254
**R_{int}**	0.0494
**Goodness of Fit**	1.039
**Parameters**	806
**Restraints**	45
**Largest Peak/Hole (eÅ^{-3})**	0.80 / -0.82
**R_1 (observed/all)**	0.0369 / 0.0499
**wR_2 (observed/all)**	0.0894 / 0.0949
**CCDC Number**	2483326

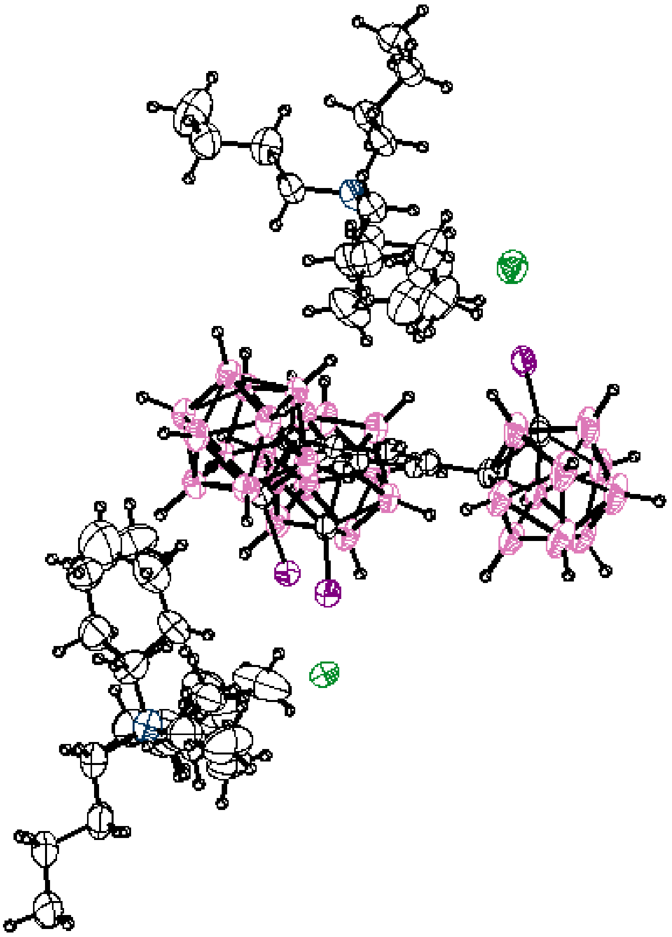

Cocrystal of the tridentate receptor and MeMIM-Br

Table S4 Crystal structure data.

**Identifier**	4
**Formula**	$C_{12}H_{33}B_{30}Br_{0.54}I_{3.46}$
**Molecular Mass [gmol^{-1}]**	983.83
**Crystal Habit**	Colorless block
**Crystal Dimensions**	0.11 × 0.13 × 0.52
**Crystal System**	Monoclinic
**Space Group**	*C*2/*m*
**a [Å]**	21.2085(10)
**b [Å]**	17.9849(11)
**c [Å]**	11.7831(7)
**α [°]**	90
**β [°]**	100.647(5)
**γ [°]**	90
**Volume [Å^3]**	4417.1(4)
**Z**	4
**Density [gcm^{-3}]**	1.479
***F*(000)**	1829
**μ [mm^{-1}]**	2.947
**T [K]**	170
**Completeness / Θ_{max}**	99.9 / 27.103
**Total Reflections**	15675
**Unique Reflections**	5026
**Reflections [Io > 2σ(Io)]**	3929
**R_{int}**	0.0592
**Goodness of Fit**	1.036
**Parameters**	222
**Restraints**	0
**Largest Peak/Hole (eÅ^{-3})**	1.59 / -1.22
**R_1 (observed/all)**	0.0536 / 0.0706
**wR_2 (observed/all)**	0.1377 / 0.1601
**CCDC Number**	2483327

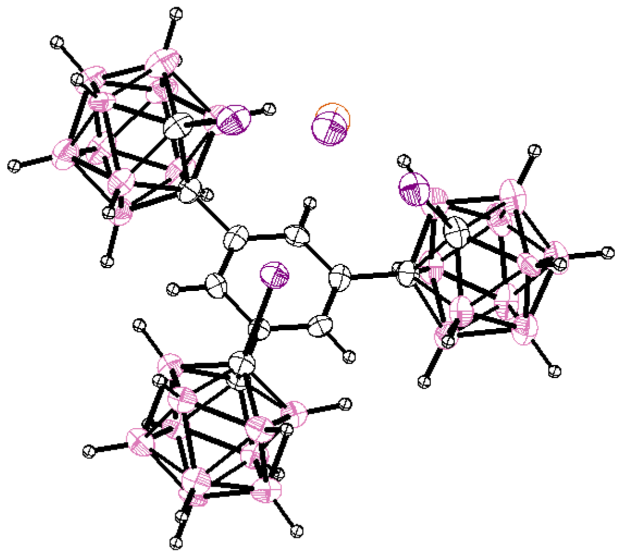

* Platon Squeeze was used to account for the cation and unknown solvate(s) present, and no correction has been made to the formula, density, *etc*.

Cocrystal of the tridentate receptor and MeMIM-I

Table S5 Crystal structure data.

**Identifier**	5
**Formula**	$C_{68}H_{168}B_{120}I_{16}N_8$
**Molecular Mass [gmol^{-1}]**	4425.69
**Crystal Habit**	Colorless needle
**Crystal Dimensions**	0.03 × 0.03 × 0.08
**Crystal System**	Monoclinic
**Space Group**	*C*2/*m*
**a [Å]**	21.3971(2)
**b [Å]**	17.8712(2)
**c [Å]**	11.79160(10)
**α [°]**	90
**β [°]**	99.6510(10)
**γ [°]**	90
**Volume [Å^3]**	4445.20(8)
**Z**	1
**Density [gcm^{-3}]**	1.653
***F*(000)**	2080
**μ [mm^{-1}]**	22.131
**T [K]**	170
**Completeness / Θ_{max}**	99.9 / 67.684
**Total Reflections**	15759
**Unique Reflections**	4544
**Reflections [Io > 2σ(Io)]**	4200
**R_{int}**	0.0283
**Goodness of Fit**	1.116
**Parameters**	240
**Restraints**	1
**Largest Peak/Hole (eÅ^{-3})**	1.48 / -1.01
**R_1 (observed/all)**	0.0296 / 0.0322
**wR_2 (observed/all)**	0.0815 / 0.0893
**CCDC Number**	2483328

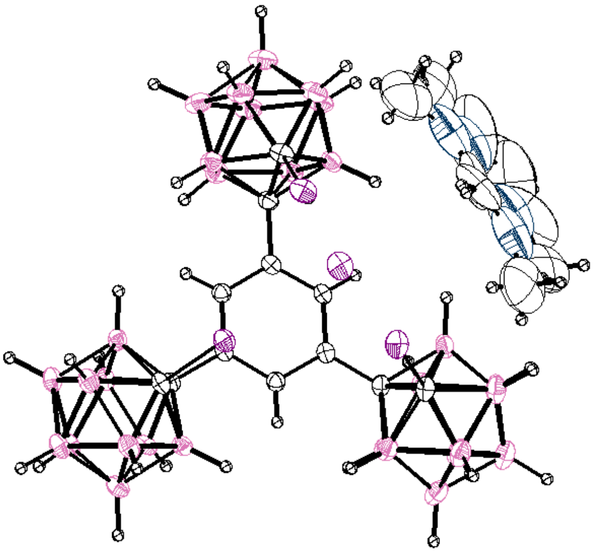

Cocrystal of the tridentate receptor and TDA-Br

Table S6 Crystal structure data.

**Identifier**	6
**Formula**	$C_{42}H_{102}B_{60}Br_{1.1}I_{6.9}N_6$
**Molecular Mass [gmol^{-1}]**	2303.40
**Crystal Habit**	Colorless block
**Crystal Dimensions**	0.14 × 0.46 × 0.59
**Crystal System**	Triclinic
**Space Group**	$P\bar{1}$
**a [Å]**	13.8770(3)
**b [Å]**	18.8975(2)
**c [Å]**	18.9117(3)
**α [°]**	97.5175(12)
**β [°]**	102.7495(16)
**γ [°]**	96.5456(14)
**Volume [Å^3]**	4742.91(14)
**Z**	2
**Density [gcm^{-3}]**	1.613
***F*(000)**	2200
**μ [mm^{-1}]**	18.519
**T [K]**	100
**Completeness / Θ_{max}**	99.9 / 66.499
**Total Reflections**	81650
**Unique Reflections**	16725
**Reflections [Io > 2σ(Io)]**	14544
**R_{int}**	0.1237
**Goodness of Fit**	1.092
**Parameters**	1046
**Restraints**	216
**Largest Peak/Hole (eÅ^{-3})**	1.77 / -2.27
**R_1 (observed/all)**	0.0630 / 0.0689
**wR_2 (observed/all)**	0.1816 / 0.1928
**CCDC Number**	2483329

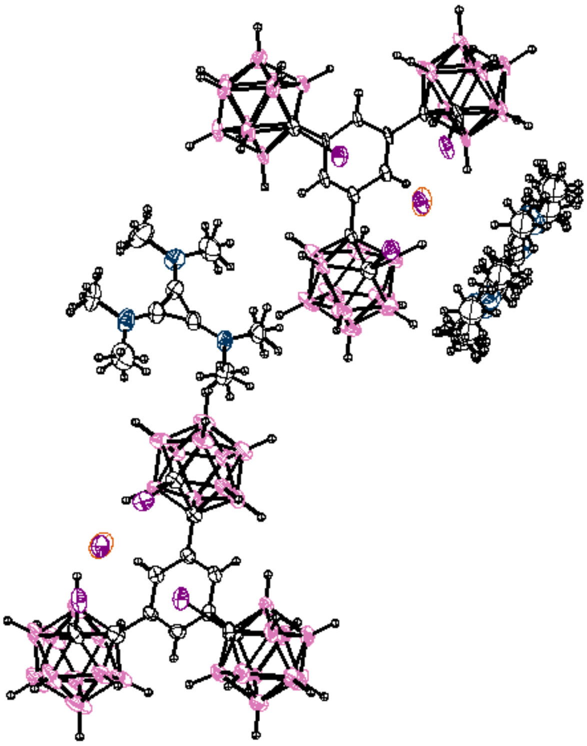

Cocrystal of the tridentate receptor and ½ MeMIM-I

Table S7 Crystal structure data.

**Identifier**	7
**Formula**	$C_{24}H_{66}B_{60}I_7$
**Molecular Mass [gmol^{-1}]**	1891.66
**Crystal Habit**	Colorless block
**Crystal Dimensions**	0.04 × 0.06 × 0.09
**Crystal System**	Monoclinic
**Space Group**	*I*2/*m*
**a [Å]**	17.1802(3)
**b [Å]**	20.2432(6)
**c [Å]**	25.0140(6)
**α [°]**	90
**β [°]**	101.157(2)
**γ [°]**	90
**Volume [Å^3]**	8535.0(4)
**Z**	4
**Density [gcm^{-3}]**	1.472
***F*(000)**	2201
**μ [mm^{-1}]**	20.133
**T [K]**	170
**Completeness / Θ_{max}**	99.7 /74.501
**Total Reflections**	55405
**Unique Reflections**	8975
**Reflections [Io > 2σ(Io)]**	6218
**R_{int}**	0.075
**Goodness of Fit**	1.006
**Parameters**	433
**Restraints**	0
**Largest Peak/Hole (eÅ^{-3})**	1.05 / -0.78
**R_1 (observed/all)**	0.0494 / 0.0736
**wR_2 (observed/all)**	0.1331 / 0.1474
**CCDC Number**	2483330

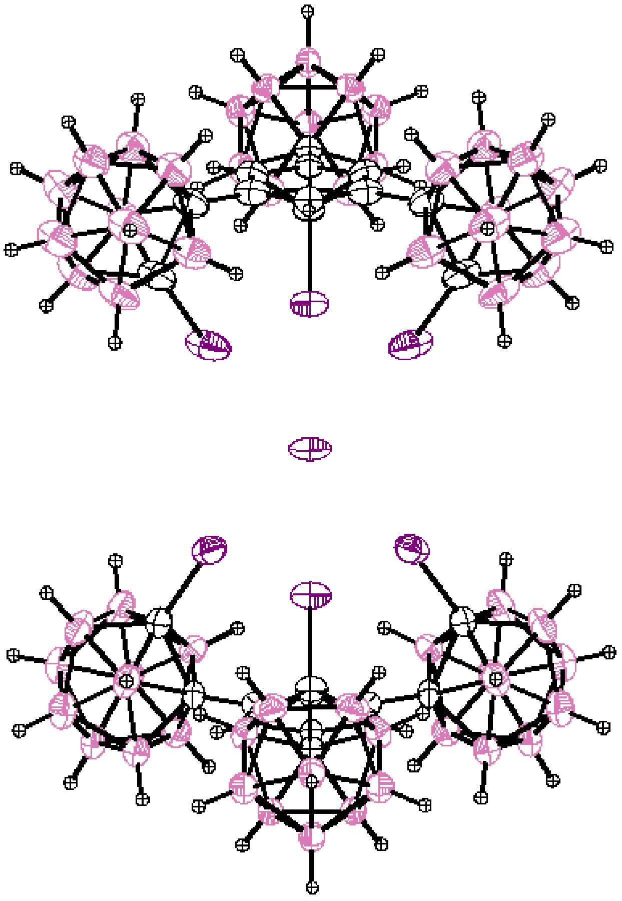

* Platon Squeeze was used to account for the cation and unknown solvate(s) present, and no correction has been made to the formula, density, *etc*.

Cocrystal of the tridentate receptor and ½ TDA-I

Table S8 Crystal structure data.

**Identifier**	8
**Formula**	$C_{35}H_{88}B_{60}Cl_4I_7N_3$
**Molecular Mass [gmol^{-1}]**	2229.78
**Crystal Habit**	Colorless block
**Crystal Dimensions**	0.04 × 0.12 × 0.21
**Crystal System**	Triclinic
**Space Group**	$P\bar{1}$
**a [Å]**	12.2486(3)
**b [Å]**	13.2480(3)
**c [Å]**	14.4882(3)
**α [°]**	105.158(2)
**β [°]**	100.478(2)
**γ [°]**	96.327(2)
**Volume [Å^3]**	2200.32(9)
**Z**	1
**Density [gcm^{-3}]**	1.683
***F*(000)**	1058
**μ [mm^{-1}]**	20.706
**T [K]**	100
**Completeness / Θ_{max}**	99.9 / 66.499
**Total Reflections**	38245
**Unique Reflections**	8839
**Reflections [Io > 2σ(Io)]**	7670
**R_{int}**	0.0573
**Goodness of Fit**	1.029
**Parameters**	585
**Restraints**	48
**Largest Peak/Hole (eÅ^{-3})**	1.62 / -1.14
**R_1 (observed/all)**	0.0390 / 0.0460
**wR_2 (observed/all)**	0.1024 / 0.1091
**CCDC Number**	2483331

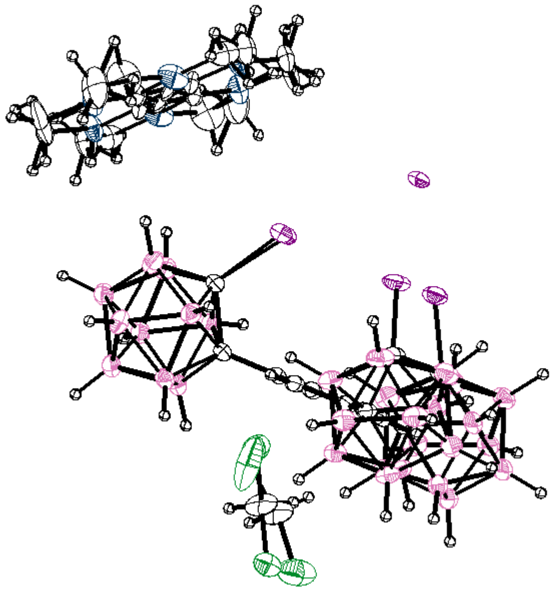

NMR

Nuclear magnetic resonance spectra were recorded on an *Avance Neo-400* (^{1}H: 400 MHz) spectrometer by *Bruker* at 30.00 °C/303.15 K. All shift values are given in ppm.

Due to the small scale of experiments, no sensible data of ^{13}C nuclei could be obtained.

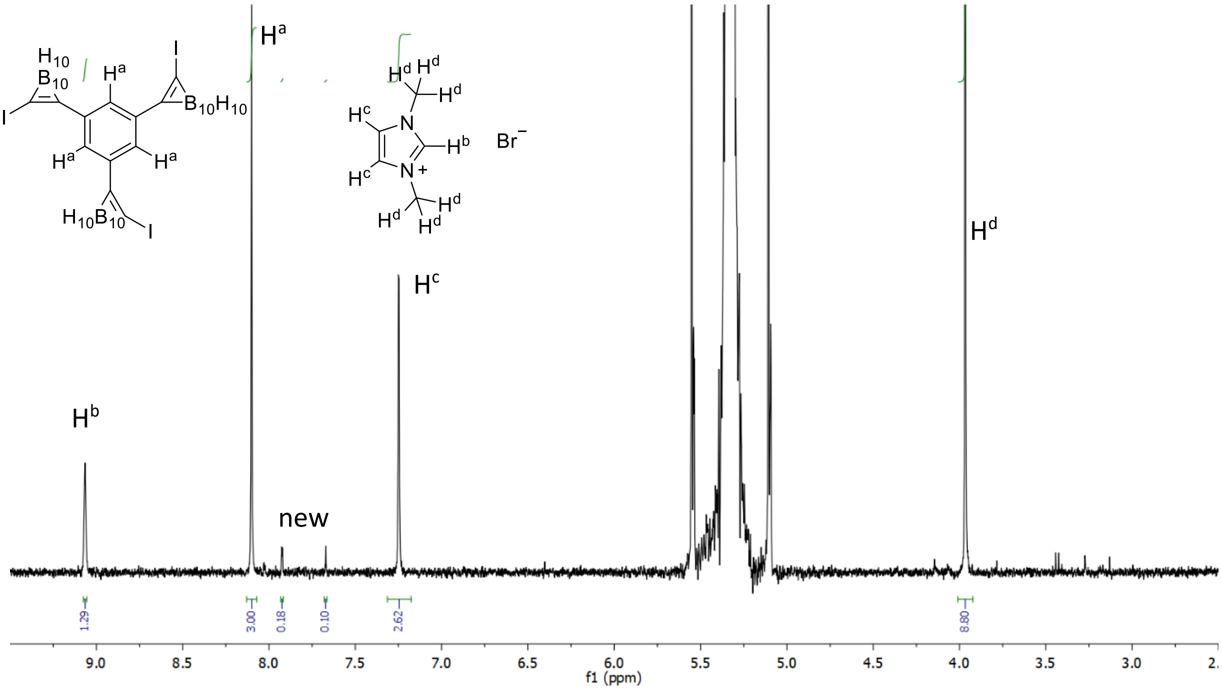

Figure S1: ^{1}H NMR (400 MHz) spectrum of redissolved crystallization sample **4** containing 1:1 of compound **1** and MeMIMBr in CD_2Cl_2. Two minor new signals are seen at 7.67 ppm and 7.93 ppm, possibly from decomposition, which might have liberated the observed iodide ions.

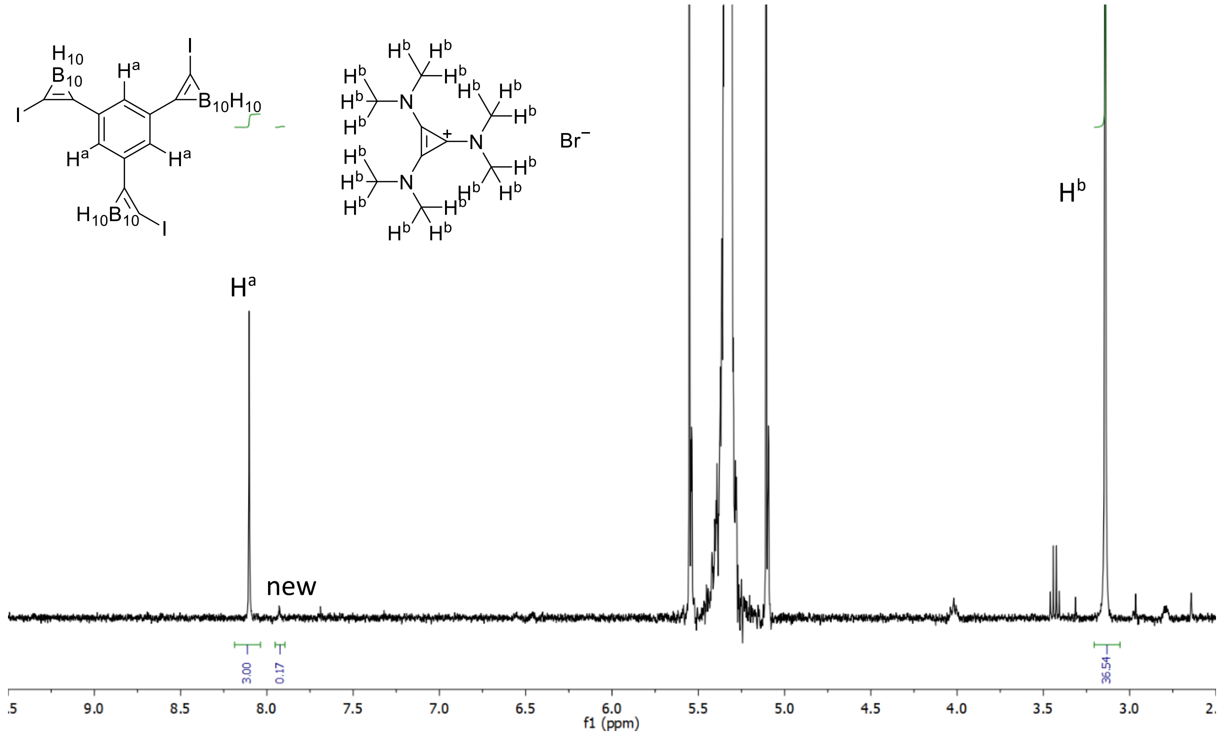

Figure S2: ^{1}H NMR (400 MHz) spectrum of redissolved crystallization sample **6** containing 1:1 **1** and TDABr in CD_2Cl_2. A minor new signal is seen at 7.93 ppm, possibly from decomposition, which might have liberated the observed iodide ions.
